# Surgeon General

**Published:** 1864-01

**Authors:** 


Surgeon General.—The *Star* says: "The President has ordered a court martial for the trial of Surgeon-General Hammond, under charges of fraud and malpractices, brought by the commission, that not long since examined into the affairs of his office.

It is publicly known, that a number of persons claiming to represent the science and philanthrophy of the country, have been exerting themselves here as a committee to produce a free judgment of the authorities against the integrity of the action of the commission instituting.

The charges for, as well as against the characters of its individual members, we may not improperly add, that Profs. Agassiz and Hierce, whose names are found among the alleged signers of the paper or address, have already taken occasion to repudiate it, upon the grounds that they never designed that their names should be used in any such manner, or for any such purpose as those of which the manager or managers of the effort to prevent due investigation of the Surgeon-General's official conduct have undertaken to use them."

The English of the above paragraph is not very manifest; but if it means anything, shows intense partizan strife, and confirms us in the opinion, that the Surgeon-General is being used, by several parties, *very meanly.*

